# Gut Microbiome Transplants and Their Health Impacts across Species

**DOI:** 10.3390/microorganisms11061488

**Published:** 2023-06-03

**Authors:** Benjamin H. Levine, Jessica M. Hoffman

**Affiliations:** Department of Biological Sciences, Augusta University, Augusta, GA 30912, USA; blevine@augusta.edu

**Keywords:** microbiome, health, transplants, dysbiosis, disease

## Abstract

The human gut, required for ingesting and processing food, extracting nutrients, and excreting waste, is made up of not just human tissue but also trillions of microbes that are responsible for many health-promoting functions. However, this gut microbiome is also associated with multiple diseases and negative health outcomes, many of which do not have a cure or treatment. One potential mechanism to alleviate these negative health effects caused by the microbiome is the use of microbiome transplants. Here, we briefly review the gut’s functional relationships in laboratory model systems and humans, with a focus on the different diseases they directly affect. We then provide an overview of the history of microbiome transplants and their use in multiple diseases including Alzheimer’s disease, Parkinson’s disease, as well as *Clostridioides difficile* infections, and irritable bowel syndrome. We finally provide insights into areas of research in which microbiome transplant research is lacking, but that simultaneously may provide significant health improvements, including age-related neurodegenerative diseases.

## 1. Introduction

The gut is one of the largest organs in the human body and is composed of both human and microbial cells. These microbes total more than 100 trillion individuals, making up a significant portion of the total cells in the human body [1,2], and they are involved in multiple physiological processes including digestion, immunity, and neurological function. While the gut microbiome contains fungi and viruses, the majority of the microbiome comprises bacteria, and variations in bacterial species and relative bacteria abundances can cause multiple health outcomes, both positive and negative. Therefore, it is imperative to understand the composition and consequences of the microbial populations in the gut [3], and discovering those individual bacteria with negative health effects is of utmost importance in the biomedical field.

Manipulating individual microbiomes has the potential to provide new treatment options for patients with different gut-associated diseases, and one of the most recently studied methods for changing microbial composition is fecal microbiome transplants (FMTs). These transplants replace the gut microbiome of a diseased individual with the microbiome of a healthy person, and ideally, the healthy microbiome colonizes the gut of the diseased individual. Rudimentary microbiome transplants have been used for hundreds of years in early medicine, but it is only recently that they have garnered widespread attention in the biomedical community [4]. Currently, FMTs are most often used to treat patients with recurrent *Clostridium difficile* infections, which can lead to colitis [5]. However, the long-term safety of FMTs is still not fully known, as there is a potential for increased risk of infection by pathogenic organisms or multidrug-resistant organisms, and this has led to the FDA halting multiple FMT clinical trials [6].

The study of the gut and FMTs has been predominately completed in the laboratory organisms *Drosophila melanogaster* and *Mus musculus*. *D. melanogaster* has a comparably simple gastrointestinal tract, making it easy to understand the direct effects of the manipulation of individual bacteria. The mouse microbiome is similar to humans and shows similar age-related variation, making it an ideal organism to understand the role of the microbiome and its health effects with direct translational potential to humans [7].

In this brief review, we investigate how the microbiomes of commonly studied laboratory species, *D. melanogaster* and *M. musculus*, compare to *Homo sapiens* regarding bacterial composition, gut structure, and differences in digestion strategies. We then discuss how the microbiome can affect organismal health. Finally, we discuss the history of microbiome transplants to improve health across species, as well as provide insights into gaps in our knowledge on the microbiome, transplants, and organismal health.

## 2. Microbiome and Gut Structure of *D. melanogaster*, *M. musculus*, and *H. sapiens*

All animals have a gut microbiome, and perhaps not surprisingly, they vary significantly across species. In a study of 54 mammalian species, no two species had an identical bacterial composition; however, some similarities were observed in species that were related by either phylogeny, gut morphology, diet, or whether the animal lived in captivity or not [8]. As with most biomedical research, the majority of microbiome studies have been completed in laboratory animals and humans. In this section, we describe the natural gut composition of *D. melanogaster*, *M. musculus*, and *H. sapiens*, as well as their similarities and differences.

**Figure 1:**
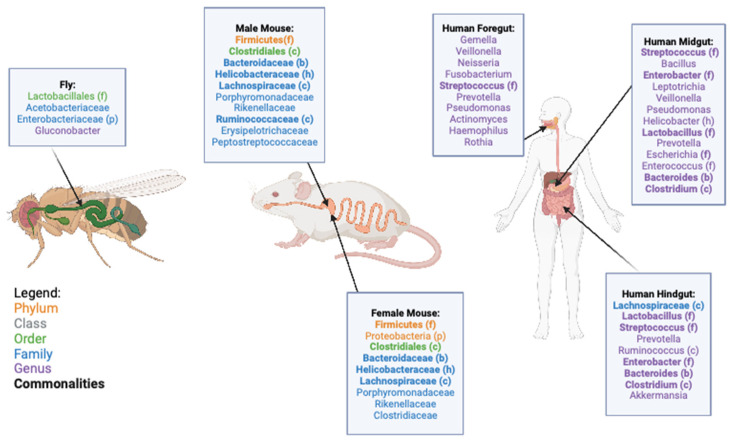
Representation of the most common Phylum, Class Orders, Families, and Genera present in the gut microbiome of *D. melanogaster*, *M. musculus*, and *H. sapiens*. Common groups are bolded between species and sections of the gut, and the phylogenetic relationship of the groups is also represented. The *D. melanogaster* microbiome is shown as a whole, while the *M. musculus* shows the differences between male and female microbiomes, and in *H. sapiens,* the different compositions across portions of the gut are shown. An (f) indicates taxa falling under the *Firmicutes* phylum, (p) for the *Proteobacteria* phylum, (c) for the Clostridiales order, (b) for the *Bacteroidaceae* family, and (h) for the *Helicobacteraceae* family [7,9,10,11,12,25]. Created with BioRender.com (accessed on 14 March 2023).

Generally, insect gut diversity is lower than mammalian gut diversity with ten to hundreds of bacterial taxa in insects compared to thousands of bacterial taxa in mammals. *D. melanogaster* is no exception, with most individuals in the species having no more than thirty bacterial taxa in their gut [9]. While the reason for this significantly lower diversity is not explicitly known, it is thought that the transient nature of insect guts compared to mammalian guts causes a reduction in diversity. This transient nature is driven by multiple factors including the short life span of the fly, the physiology of the gut where portions of the foregut and hindgut are shed during molting, as well as the entire larval gut being replaced with an adult gut during metamorphosis. [9]. *D. melanogaster* lab microbiomes are most commonly composed of four taxa of bacteria (Figure 1); however, the composition of wild flies is more variable, with lower abundances of the taxa seen in lab-reared flies and higher numbers of unidentified taxa [9,10,11,12]. Other than the four major taxa, lab-reared flies can contain other gut microbiome taxa, but they vary across strains and are present in relatively low percentages [11]. These taxa are made up of 31 additional families and 18 different orders, all with amounts less than 1% of the total bacterial microbiome, with several of these taxa being known symbionts of other animals such as the *Clostridiales, Bacteroidales,* and *Actinomycetales* orders [10]. Sex differences have not been well established in natural populations of flies, but there are significant sex differences in response to dietary changes that appear to be driven by the microbiome [13].

*D. melanogaster,* and insects in general, acquire their gut microbes from their environment rather than through vertical transmission—the direct acquisition of microbes from mother to offspring, which is described later in the paper [14]—though there is evidence of some vertical transmission effects of the microbiome on offspring [15]. When *D. melanogaster* is in its embryonic state, it is sterile, and only acquires its microbiome by consuming the feces of adult flies, as well as any bacteria that are present in their food [9,16]. Small changes to the fly diet can have large effects on the microbiome composition. For example, the addition of methylparaben, a commonly used anti-fungal agent, to *Drosophila* media can shift the microbiome of flies [17]. In addition, high fat or starvation diets significantly change the *D. melanogaster* microbiome [18]. This indicates that the bacterial composition of *D. melanogaster*’s microbiome could be controlled to some extent by the food that they feed on, and they may provide an ideal model system to study the effects of dietary bacterial changes on health. However, there is significant variation within “standard” diets in the *Drosophila* field, which can lead to conflicting results in microbiome analyses [19]. Therefore, comparing the results of microbiome studies and the microbial community’s effect on health can be difficult across *Drosophila* studies.

As mammals, *M. musculus* microbiomes are more diverse than those of *D. melanogaster* and are more likely to vary depending on diets and physical location, rather than between strains [7,20,21]. There are seven prominent taxa of bacteria in laboratory mice (Figure 1)*,* and there are significant sex differences in the microbiome between male and female mice (Figure 1, [7]). However, there are numerous unidentified bacterial taxa in the mouse microbiome that, once identified, might constitute a larger portion of the gut than those described in Figure 1. Controlling the microbiome composition of *M. musculus* is more difficult than *D. melanogaster,* as their microbiomes are less transient. For this reason, microbiome repopulation usually requires using broad-spectrum antibiotics to wipe out the existing microbes before any new bacteria can be introduced. Clearing the microbiome in this way has some drawbacks, including not completely removing the bacteria in the gut, allowing for fungal overgrowth, reducing important bacterial populations outside the gut, and, as with any antibiotic treatment, the potential to develop antibiotic-resistant bacteria [22]. As the microbiome has very important physiological functions in an individual, employing methods to manipulate the microbiome, without completely removing the starting microbiome, is required. Additionally, similar to *Drosophila* studies, dietary manipulations may be a viable option to shift microbiome compositions. Mice fed a high-fat diet have a decrease in *Ruminococcaceae* and an increase in *Rikenellaceae* in their microbiome [23]. The increase in *Rikenellaceae* is of particular interest as *Alistipes*, a genus within *Rikenellaceae*, has been associated with type-2 diabetes in humans [23,24]. Reducing specific taxa of bacteria via dietary manipulations is potentially a more viable option for human translational studies as well.

**Figure 2:**
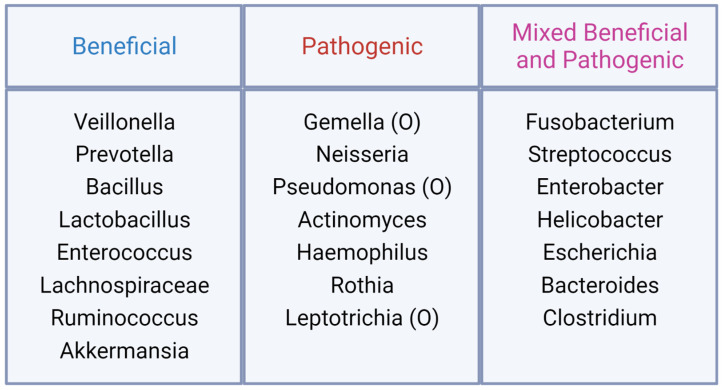
Most common genera in the gut microbiome of *H. sapiens*, and their relationships to human health. *Lachnospiraceae* is an exception, as it is a bacterial family. An (O) indicates that the genera are opportunistic pathogens [27,28,29,30,31,32,33,34,35,36,37,38,39,40,41,42,43,44,45,46,47,48,49]. Created with BioRender.com (accessed on 11 May 2023).

The microbial composition of the *H. Sapiens* microbiome is, perhaps not surprisingly, much more diverse than other mammalian species, with additional variation across different sections of the gut. The human microbiome is vertically transmitted from the mother, and in early life is dominated by the *Bifidobacterium* genus and the *Bacteroides* genus. During the adolescent years, the *Bifidobacterium* and *Faecalibacterium* genera and the *Lachnospiraceae* family dominate before bacterial taxa diversity increases with age [25]. During adulthood, the microbiome settles into five different “minibiomes” in the oral cavity, esophagus, stomach, small intestine, and the colon. There are 21 primary taxa that occupy each of these minibiomes in different combinations (Figure 1), including many more taxa that only represent a small portion of the microbiome [25]. It is estimated using gene cataloguing that there are over 1000 distinct bacterial species at any one time that occupy the *H. sapiens* gut, with only 57 of those species common to over 90% of all individuals and 75 species common to over 50% of all individuals [26,27,28,29,30,31,32,33,34,35,36,37,38,39,40,41,42,43,44,45,46,47,48,49]. In addition, these bacterial species can be beneficial, pathogenic, as well as a mixture of both depending on the study (Figure 2). Thus, it is obvious that there is a large variation in the microbiome across individuals. In humans, differences between males and females are unclear. Generally, females have a higher α-diversity, which refers to the number of species and related abundances within a sample, but specific genera and species are extremely variable [50]. As with *M. musculus,* the *H. sapiens* microbiome varies with age, geographic location, and diet, but it additionally varies based on the ethnic background and socioeconomic status of an individual [51,52]. In data from the American Gut Project, 12 microbial genera and families varied with ethnicity and were more strongly associated with ethnicity than BMI, age, or sex. Similarly, the *Christensenellaceae* family, which had previously been associated with BMI, was not associated with BMI across all ethnicities [52,53,54]. Socioeconomic status was also linked to the gut microbiome with a higher α-diversity and abundance of *Bacteroides* found in individuals of higher socioeconomic status [52,55,56]. Overall, the human microbiome is significantly influenced by many external factors, making comparisons across populations and studies difficult.

*D. melanogaster, M. musculus,* and *H. sapiens* gut structures are generally similar with three main sections: initial ingestion of food, digestion of food, and final digestion and excretion of waste. Specifically, the *D. melanogaster* gut contains a foregut, which includes the oral cavity, esophagus, crop, and cardia. The crop is unique to *Diptera* and thought to be involved in early digestion, detoxification, microbial control, and food storage, and the cardia is the site of antimicrobial peptide production and regulation of food entry into the midgut [57,58]. The *Drosophila* midgut is similar to the human and mouse stomach, and the hind gut is similar to the human and mouse small and large intestine [58,59]. There has also been recent evidence that the *Drosophila* midgut is more similar to the human and mouse midgut than previously thought. Most insects have a midgut epithelial polarity that is inverse compared to humans and mice, but in the midgut of *Drosophila*, the polarity switches, making it more similar to humans [60]. In addition to this basic gut structure, there is evidence of extraoral digestion in *D. melanogaster*, where saliva is excreted onto food before the fly consumes it, aiding in the initial digestion [57].

As they are both mammals, the gut structures of *M. musculus* and *H. sapiens* are, not surprisingly, more similar to each other than they are to *D. melanogaster*. Mammalian guts have the same general structure with the oral cavity leading to the esophagus, then the stomach, small intestine, cecum, large intestine, and colon in the order of digestion [21,61]. The primary difference between the mouse and human gut is that *M. musculus* has significantly longer small and large intestines by body weight than *H. sapiens,* and the mouse colon produces pelleted feces compared to segmented feces in humans. The relatively longer intestine allows mice to digest their food more quickly than humans, while still extracting all the necessary nutrients and energy, which is shown by mice having an intestinal transit time of 6–7 h versus the human transit time of 14–76 h. Lastly, *Muridae* have a forestomach that stores food for extended periods of time until energetic needs trigger its release, similar to the crop in *D. melanogaster* [21,62].

## 3. Gut Microbiome and Health

As stated above, the gut microbiome is composed of a variety of different microorganisms, including bacteria, fungi, viruses, and archaea that exist within the digestive systems of animals. These microorganisms can provide either symbiotic, neutral, or pathogenic effects to the host animal [63]. In humans, for example, gut microorganisms are the main synthesizer of vitamin B and vitamin K, as well as the main metabolizers of bile acids, sterols, and xenobiotics [64]. Generally, within a species, there is a set of microorganisms that are consistent across individuals; however, as described above, the overall composition, both in bacterial concentration as well as species diversity, of the microbiome can vary greatly between individuals. These changes can occur in response to external as well as endogenous physiological processes, including, but not limited to, aging, dietary changes, the use of antibiotics, genetic differences, and the response to infection [65,66]. Dysregulation, a state of imbalance in the composition or function of microbial taxa, of the gut microbiome can lead to significant inflammatory and autoimmune conditions [65,67]. There is a large drive to understand how these factors influence the microbiome to potentially develop interventions to prevent negative health outcomes [66,68], yet overall research in the field is still in its infancy.

One significant health effect of the gut microbiome is their effect on intestinal barrier permeability. A healthy gut allows nutrients, water, and ions to pass out of the gut and into the bloodstream while blocking pathogens and bacterial toxins from crossing the barrier [69]. However, as individuals age or are confronted with disease, their gut can become more permeable, even allowing toxic molecules and pathogens to move out of the gut and into the bloodstream where they can move systemically through the body. As the gut becomes more permeable, the tight junctions between intestinal epithelial cells weaken, which allows these previously contained molecules and bacteria to leave the gut and travel systemically in the body [69,70]. The gut microbiome can significantly affect gut permeability [71], and reducing gut permeability may have significant translational health benefits.

The gut has a variety of functions outside of the digestion and absorption of nutrients, including aiding in the production of cytokines, the maintenance of homeostasis, T-cell production, and immune system regulation [72]. Additionally, dysregulation in the gut is implicated in diseases such as inflammatory bowel disease, type 1 diabetes, multiple sclerosis, HIV, and several different cancers, which can be driven by intestinal barrier failure or declines in immune responses [72]. Almost all of these diseases are related to a disruption in normal gut function and are associated with the dysbiosis of the microbiome. Dysbiosis can refer to a few different types of microbiome dysregulation, including the loss of beneficial microbes, the overgrowth of harmful microbes, or the loss of diversity in the microbiome [73]. These dysregulations often have similar causes including serious infections of the gut, poor diet, lack of exercise, increasing age, and even an excessive use of antibiotics [73]. Understanding the causes of gut dysbiosis and dysregulation is necessary to develop novel interventions to treat gut-related health disorders and diseases.

There are strong genetic links between dysregulations of the gut and the diseases they are associated with, and one of the most informative laboratory animals available to study host genetic influences on microbiome composition is the fruit fly, *D. melanogaster.* As stated previously, the fruit fly gut contains a very limited number of bacterial taxa, and this coupled with the small genome and genomic tools available in the fly, easily allow us to understand how the interaction of the microbiome and host genome can influence health outcomes. Changes in the *Drosophila* genome have been associated with variations in gut bacterial composition, indicating that genetic variation can affect microbial abundance [74]. Most work regarding this connection has been completed in axenic flies, which do not contain a gut microbiome. Axenic flies are usually produced by rinsing *Drosophila* eggs with hypochlorite to kill any microorganisms on the surface of the eggs, before transferring the eggs onto a sterile medium [58]. For example, different strains of axenic flies exposed to monocultures of bacteria showed significant differences in the amount of the individual bacteria that could colonize the gut, indicating genetic effects of the fly on microbiome composition [74]. In addition, a genetic variation in the fly has causative effects on the microbiome that, consequently, have a direct effect on metabolic parameters associated with health, such as glycogen content, triacylglycerol storage, glycerol levels, and metabolic rate, suggesting that genotype differences can lead to interactions between physiological parameters and the microbiome [75,76]. There is also evidence for genetic effects on the microbiome in *Mus musculus* where genetic influences were responsible, on average, for 19% of the variation present in the gut microbiome composition [77]. However, caging effects and inter-individual variation made up 31.7% and 45.5% of the variation, respectively, indicating that while genetics have an effect on gut microbiome composition, they are far from the most influential factor [77]. As gut microbial composition is at least partially driven by host genotype, the potential exists for the treatment of gut-related health disorders by targeting gene therapies to the host, and future research is needed to completely tease apart these host-gene-by-microbiome interactions.

The abundance of health-associated bacteria and bacterial composition stability has also been shown to be transmissible between parent and offspring across species. This transmission of the microbiome to offspring can be the result of a few different processes; however, it is mainly the result of three primary routes: genotype inheritance, parental inheritance, and environmental inheritance, though parental inheritance can look very different between different species. Genotype inheritance is the transmission of genes from parent to offspring in which the genes affect which microbes are destroyed as well as which are allowed to divide and grow. This drives the genetic effects described above in flies and mice. In humans, this genetic control helps to shape the microbiome appropriately for physiological homeostasis. For example, before infants eat solid food, their microbiomes are enriched with microbes that help to utilize the lactate that they are consuming high levels of, but once solid food is consumed, the microbiome becomes more “adult-like”, enriched with microbes that can utilize carbon, biosynthesize vitamins, and degrade xenobiotics [78]. The changes to a more “adult-like” microbiome are mediated by the genetics of the child combined with these dietary changes. Heritability of the abundance of health-associated bacteria, bacterial composition stability, and disease phenotypes along the gut–brain axis has also been described [79,80,81,82]. For example, the *Christensenellaceae* family has been shown to be highly heritable in humans, with an average of 40 to 60% heritability [82], and the *Christensenellaceae* family is composed of beneficial bacteria that are found in higher concentrations in individuals that have a lean body mass index [82]. Overall, it is obvious that host genomic variation can influence microbial variation, though more research on the individual genes that affect these differences is still required.

A common method of understanding the genetic and environmental effects of microbiome transmission in humans is through twin studies. Generally, in twin studies, the microbiome compositions correlate and persist even if the environment of the twins differ, with the most genetically heritable traits including microbial abundance and the relative abundance of different microbial taxa [80]. Some examples of genetic links between the gut microbiome composition and individual diseases have also been discovered in non-twin studies using large genetic databases. For example, eight genes affecting the microbiome have been associated with schizophrenia [81], and significant alterations in the gut microbiome have been observed in individuals with a high genetic risk for irritable bowel disease (IBD) before the clinical manifestation of the disease. Specifically, the *Roseburia* genus has been tightly associated with the risk to develop genetic IBD [83]. These genetic links indicate that the human genotype can have a significant effect on the composition of the gut microbiome, which then itself affects disease severity and prevalence, though more research in the field is still required.

Parental inheritance is the direct passage of bacteria from mother to offspring. This process varies between mammals and non-mammals, where in mammals, bacteria colonize the offspring both in the womb and through breast milk, while in non-mammals, the embryos are colonized with microbes from the mother before being laid. In contrast, certain species such as *D. melanogaster* do not have any parental inheritance, and eggs are sterile when laid, acquiring their microbiome from their parents indirectly through the environment [58,78,84]. With environmental inheritance, individuals living in the same environment have a similar microbiome, as they are exposed to the same local bacteria. This could be the direct result of individuals leaving feces or other stomach contents in the environment that the offspring ingest, or as in *Drosophila* where environmental bacteria colonize the surface of the egg shell that then enter the gut of the larva as it emerges from the egg [58]. These three modes of microbiome inheritance provide the foundation for the wide diversity that is seen in microbiomes, and understanding the transmission of microbiomes may help us develop novel interventions to gut-associated diseases.

Interestingly, the gut microbiome can also significantly impact the brain. This connection between the gut and brain, often referred to as the gut–brain axis, is tightly regulated, and a dysfunction in one can lead to a dysfunction in the other [85]. This communication system is bidirectional, and dysbiosis of the gut has been associated with diseases falling into two main categories, gut diseases affected by the central nervous system and central nervous system diseases affected by the gut. The former include IBD, functional constipation, diarrhea, and fecal incontinence, and the latter include depression, anxiety, stress, and autism [3,86]. To study these diseases, it is important that the microbiome be tightly controlled, to minimize the progression and severity of diseases. To this end, gut–brain axis disorders have primarily been analyzed in germ-free mice. Germ-free mice contain no gut microbiome and are kept in strict barrier facilities to prevent the introduction of any foreign bacteria, similar to axenic flies. By ensuring the germ-free mice are not introduced to any foreign bacteria, they allow us to analyze the effect of removing the microbiome on specific diseases, as well as understand what happens when microbes are reintroduced into their guts. Reintroduction experiments include recolonization and microbiome transplant experiments, where the gut microbiome of another non-germ-free individual is donated to the germ-free individuals. These transplant methods are developed with the hope to reverse, entirely or temporarily, disease phenotypes associated with dysbiosis. For example, germ-free mice have increased motor activity and reduced anxiety-like responses compared to mice with a normal gut microbiome, and these effects were also observed to be reversed by the colonization of the germ-free mice gut with a normal microbiome early in life [3,87], suggesting that the microbiome can influence the brain and neuronal responses. It has also been shown that when transplanting the gut microbiome of both young and old mice to young germ-free mice, both mice experience positive weight gain and an increase in skeletal muscle mass [88]. Additionally, the germ-free mice receiving a donation from old mice showed increased neurogenesis in the hippocampus and increased intestinal growth, driven by an enrichment of butyrate-producing bacteria including *Lachnospiraceae* and *Firmicutes*, suggesting that not all aging effects are negative [88]. As might be more expected, young germ-free mice transplanted with old mouse microbiomes also saw energy imbalances and a decline in their stress recognition systems [88]. However, it should be noted that while germ-free mice are a powerful model for studying direct effects of the microbiome, the lack of environmental variation and natural microbiomes make comparisons to humans difficult.

## 4. Microbiome Transplants

While studies into the gut microbiome are a more contemporary subject, microbiome transplants, also known as fecal microbiota transplantations (FMTs), have been performed in medicine for centuries. The first records of an attempted fecal transplantation come from 4th century China where a man named Hong Ge treated patients suffering from severe food poisoning and diarrhea with “yellow soup”, which contained fecal matter and water to be drunk by his patients [4,89]. This practice continued into the 16th century, and a large variety of fecal-based products were developed to treat assorted gastrointestinal complaints, as well as fever and pain [89]. The first official clinical trial in humans was completed in 1958, to treat four patients with fulminant pseudomembranous colitis, which is the result of a severe *C. difficile* infection that causes acute inflammation of the colon and systemic toxicity. These patients were treated with a fecal enema, and all four patients were deemed healthy, with no symptoms of infection, shortly after the enema was completed [90,91]. Since then, FMT has been used to treat ulcerative colitis, constipation, diarrhea, abdominal pain, Crohn’s disease, and *C. difficile* infections all with generally high effectiveness [92]. FMTs are delivered via multiple routes, depending on where colonization is intended to occur, with oral capsules, upper endoscopies, and nasoenteric tubes used for the foregut and midgut, and colonoscopies being used for the hindgut. FMTs are typically recommended when antibiotics no longer work on an infection, and toxicity only tends to occur when a donor has pathogenic or multidrug-resistant microbes present in their stool. A sign of early success, the first clinical trial analyzing the treatment of *C. difficile* ended early due to its effectiveness of over 81% with a first dose and 90% with a second dose, such that it was “harmful” to the controls to not receive the treatment [92,93,94]. This study provided strong evidence that FMTs had a high potential for treatment of gut diseases and needed further exploration in the medical community.

In recent years, researchers have attempted to develop alternatives to fecal transplants, as direct fecal transplants are associated with multiple negative side-effects including transient diarrhea, abdominal cramps or pain, low-grade fever, bloating, flatulence, and constipation [95]. Rectal bacteriotherapy uses cultured strains of bacteria originating from human feces and then transplants a set of those cultured bacteria into the patient’s gut [96]. This procedure resulted in an approximate 60% recovery from *C. difficile* and could be a powerful alternative to true FMT [96], as only a couple bacteria are transplanted, not an entire microbiome.

FMTs are also quite common in the veterinary world, especially in ruminants where they are used to treat simple indigestion by recolonizing their gastrointestinal tracts with healthy bacteria [97]. They are also common in nature with many animals participating in coprophagia, including elephants, hippos, koalas, and pandas [97]. Coprophagia is thought to be a way to gain extra nutrients from fecal waste; however, it also causes transfers of bacteria into the gut [98]. Some coprophagic animals must consume the feces of either their parents or other individuals in their environment, as they are born with sterile gastrointestinal tracts [97]. Consuming the feces in the wild might be considered the most basic fecal transplant, allowing the bacteria present in the feces of one individual to colonize the gut of a second individual, so that the recipient can develop proper digestion [97]. While wild animals can be good subjects for studies into microbiome transplants, ensuring control and exposure to a specific microbiome of interest can only be completed in laboratory settings.

Even though *D. melanogaster* has the simplest and easiest-to-study microbiome, they arguably have had microbiome transplants explored the least, with the majority of studies opting to make minor changes to the microbiome of the flies rather than replacing it or altering it in a major way. The human microbiome has been transplanted into the flies with between 80 and 87.5% of the bacterial taxa in the donor feces being incorporated into the *Drosophila* microbiome. The flies were exposed to the fecal material of the donor and were observed for 36 days following the exposure [99]. Both male and female flies showed increased lifespan and improved climbing ability with age; however, the improvements in age-related physical function and longevity were more prominent in males compared to females [99]. Parkinson’s disease (PD), a brain disorder that causes unintended or uncontrollable movements and is associated with a loss of dopaminergic neurons in the substantia nigra, leading to dementia in late stages of the disease, has also been modeled in *D. melanogaster.* The microbiomes of the PD model gene *park^25^* flies were transferred to the larva of both control flies and *park^25^* flies, resulting in a negative effect on both pupation and eclosion, with a more significant effect on the PD model flies [100,101]. These studies show that microbiome transplants are possible in flies; however, the overall use of microbiome transplant research in flies is still limited. The fly provides an ideal model to quickly study the effects of specific microbial compositions on different health- and age-related phenotypes, and potentially these results can then be translated into other model systems and humans.

Perhaps not surprisingly, the majority of experimental microbiome transplant studies have been completed in the laboratory mouse. Previous research has shown that transplanting the microbiome of an obese human into mice causes both vascular dysfunction and glucose intolerance [102]. Conversely transferring the microbiome of a normal-weight individual into mice resulted in the animals having higher concentrations of *Bifidobacterium, Lactobacillus,* and *Bacteroides ovatis*, which are beneficial bacteria found in the human gut. In addition, the “normal-weight microbiome” mice had reduced arterial stiffness and improved glucose tolerance [102]. Along similar lines, FMTs from obese to normal mice led to increased gut permeability and other negative health outcomes [103]. Interestingly, when transferring the microbiome of young mice to old mice, phenotypes associated with older age, such as increased intestinal barrier permeability, age-associated central nervous system inflammation, retinal inflammation, and the loss of key functional eye proteins, were reversed in the short-term [104]. However, these changes, and the bacterial changes observed, returned to baseline approximately 18 days after transplantation, suggesting that for long-term benefits, consistent young-to-old FMTs will be required [104]. Conversely, when transferring the microbiome of an aged mouse to a younger mouse, spatial learning and memory were negatively impacted, showing that although the age-related differences between mice microbiomes are minor, they still can have large effects on the health of an individual [105].

As discussed earlier, humans have implemented FMTs for over 1700 years, and newer studies are only solidifying the power of FMTs in non-gut-specific diseases. A case study was performed on three patients that were diagnosed with multiple sclerosis (MS) that received FMTs to treat constipation. In all three cases, after the fecal transplant, both the constipation and other, non-gut related, MS symptoms were alleviated, including improved ambulatory function [106]. These results point again toward the systemic effects that can occur with only modulation of the gut microbiome. When considering the current literature, it is clear that FMTs have a positive benefit to individuals with specific diseases including reductions in *C. difficile* infections, ulcerative colitis, and diarrhea [92,93,94]. As these transplants are very effective at improving symptoms related to gut diseases, it then raises the question whether they could be effective in improving phenotypes not directly associated with the gut, such as aging, and neurological conditions that are linked to the gut such as Alzheimer’s disease (AD) and Parkinson’s disease (PD).

**Figure 3:**
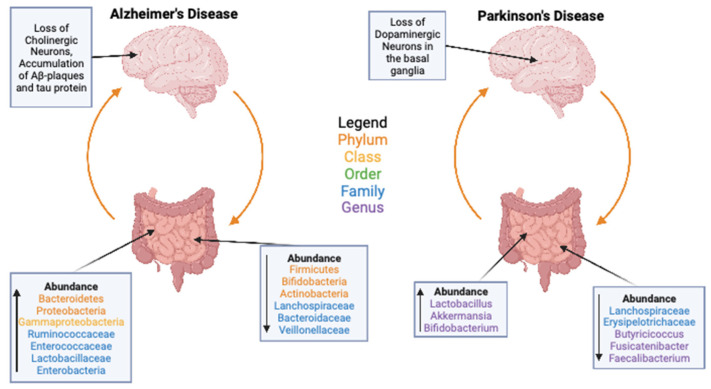
Bacteria implicated in the gut–brain axis in Parkinson’s Disease (PD) and Alzheimer’s Disease (AD). Shown for both diseases are common Phyla, Classes, Orders, Families, and Genera that are either upregulated and increase in relative abundance or that are deregulated and decrease in relative abundance [107,108,109,110,111,116,117,118]. Created with BioRender.com (accessed on).

Alzheimer’s disease (AD), even though neurological, is highly associated with the gut. AD patients have a less diverse gut microbiome than control adults of similar age [107,108,109,110,111]. It is thought that these imbalances in the gut microbiome contribute to the early stages of AD, increasing inflammation, oxidative stress, and cytokine secretion [110,112,113]. While overall diversity is lower, specific taxa of bacteria have been identified as changing in terms of abundance in individuals with AD (Figure 3). However, these results are often conflicting [107,108,109,110], and these differences are thought to be the result of differences in ethnicities based on the populations of study [110,114]. Therefore, baseline ethnic differences in the microbiome can also influence the associations of age-related and diseased microbiomes, such that more research is needed on the direct role of changes in the microbiome that are affecting AD phenotypes across populations. In a recent study, the tau-protein-mediated neurodegeneration of AD has been linked to the microbiome in mice, showing that when manipulating the microbiome with either antibiotics or germ-free rearing, a strong reduction in inflammation, tau-protein-related neurodegeneration, and brain atrophy in the hippocampus was seen, and the effects were modulated by ApoE, the protein most associated with the development of AD in humans [115].

Even more so than AD, the Parkinson’s disease (PD) phenotype is significantly linked to the gut, with gut dysbiosis being one of the first clinical manifestations that PD patients present. Perhaps not surprisingly, there are also significant changes in the microbiome in PD patients (Figure 3, [116,117]). When compared to the AD microbiome, the PD microbiome is significantly different in the specific bacteria present, and PD patients do not show the general abundance and diversity decrease that is present in AD [108,118]. This potentially indicates that the decrease in microbiome abundance is significant in the progression of AD but not in PD. Increased gut permeability is a common phenotype in PD and leads to gut inflammation, which is thought to be involved in the development of PD, and patients with inflammatory bowel disease have a higher risk for PD [118,119]. In mice, it has been shown that in rotenone-induced PD models, FMTs can correct the PD-induced dysbiosis as well as reduce GI disturbances and motor deficits [120]. However, it is still not well known if these microbiome changes are causative of any PD symptoms or just a byproduct effect.

As both AD and PD are tightly linked to the health of the gut microbiome, there has been substantial thought into if microbiome transplants could be an ideal intervention to slow both diseases. AD patients with *C. difficile* infections were treated with a FMT, and the treatment caused significant cognitive improvement, including improved mental acuity, memory, and mood. However, this effect was temporary as the microbiome quickly returned to its native state [121,122]. This suggests that FMTs in AD patients may be similar to the age-related results in mice where to keep a “youthful” microbiome, continuous transplantation will be required. A consistent effect is seen in PD, where PD patients with a history of constipation were treated with FMT. After treatment, the constipation cleared quickly, and the patients’ motor symptoms improved. However, as with the AD patients, this effect was lost over time as the gut microbiome returned to its normal state in the patient [123,124]. These results indicate that microbiome transplants could be an effective treatment for these diseases; however, the short-term nature of the results indicates that constant retreatment may be necessary. This idea of retreatment has also been considered in mice, where aged mice had their microbiomes cleared with antibiotics before being either untreated, treated with their own microbiome, or treated with a microbiome from another individual. It was shown that when transferring another individual’s microbiome, the microbiome of the treated mouse slowly trended back to the baseline the mouse had initially [125]. The mechanism of this interaction does not seem to have been categorized but potentially could be linked to the genotype of the diseased individual. After being inoculated with the healthy microbiome, the host’s body begins returning the microbiome back to its default state, resulting in only this transient change.

## 5. Conclusions

Overall, the gut microbiome is an essential part of human life and is implicated in multiple diseases, both gastrointestinal in origin and outside the GI tract. Therefore, having a more thorough understanding of the methods available to improve the health of the gut microbiome is imperative. While microbiome transplants from healthy individuals to sick individuals may be a viable intervention to prevent and treat multiple gut-associated diseases, we need to keep in mind the risk of transferring potentially pathogenic or multidrug-resistant bacteria between people. Additionally, as microbiomes are very diverse across individuals and populations, what works in one person may not be best for another individual, and a personalized medicine approach may be necessary. Long-term, large-scale studies of FMTs are needed to determine the viability and efficacy of these interventions across populations. In addition, there are still limited data on the role of FMTs to reverse or slow the development of age-related diseases, as well as other diseases that might be impacted by the gut. FMTs could potentially be a powerful technique to extend lifespan and improve the standard of living for older adults. Lastly, while this review focused on GI microbiome transplants, there is the potential for these transplants to be applied to microbiome populations in other organs in which dysbiosis occurs, including the skin. Overall, while FMTs have a long history, there is still a large gap in our knowledge about the potential of these medical procedures to improve health, and we are interested to see where research in the field leads over the next decade.

## Data Availability

No new data were created or analyzed in this study. Data sharing is not applicable to this article.
